# Prediction and Characterization of Small Non-Coding RNAs Related to Secondary Metabolites in *Saccharopolyspora erythraea*

**DOI:** 10.1371/journal.pone.0080676

**Published:** 2013-11-13

**Authors:** Wei-Bing Liu, Yang Shi, Li-Li Yao, Ying Zhou, Bang-Ce Ye

**Affiliations:** Laboratory of Biosystems and Microanalysis, State Key Laboratory of Bioreactor Engineering, East China University of Science and Technology, Shanghai, China; University Paris South, France

## Abstract

*Saccharopolyspora erythraea* produces a large number of secondary metabolites with biological activities, including erythromycin. Elucidation of the mechanisms through which the production of these secondary metabolites is regulated may help to identify new strategies for improved biosynthesis of erythromycin. In this paper, we describe the systematic prediction and analysis of small non-coding RNAs (sRNAs) in *S. erythraea*, with the aim to elucidate sRNA-mediated regulation of secondary metabolite biosynthesis. *In silico* and deep-sequencing technologies were applied to predict sRNAs in *S. erythraea*. Six hundred and forty-seven potential sRNA loci were identified, of which 382 *cis*-encoded antisense RNA are complementary to protein-coding regions and 265 predicted transcripts are located in intergenic regions. Six candidate sRNAs (sernc292, sernc293, sernc350, sernc351, sernc361, and sernc389) belong to four gene clusters (*tpc3*, *pke*, *pks6*, and *nrps5*) that are involved in secondary metabolite biosynthesis. Deep-sequencing data showed that the expression of all sRNAs in the strain HL3168 E3 (E3) was higher than that in NRRL23338 (M), except for sernc292 and sernc361 expression. The relative expression of six sRNAs in strain M and E3 were validated by qRT-PCR at three different time points (24, 48, and 72 h). The results showed that, at each time point, the transcription levels of sernc293, sernc350, sernc351, and sernc389 were higher in E3 than in M, with the largest difference observed at 72 h, whereas no signals for sernc292 and sernc361 were detected. sernc293, sernc350, sernc351, and sernc389 probably regulate iron transport, terpene metabolism, geosmin synthesis, and polyketide biosynthesis, respectively. The major significance of this study is the successful prediction and identification of sRNAs in genomic regions close to the secondary metabolism-related genes in *S. erythraea*. A better understanding of the sRNA-target interaction would help to elucidate the complete range of functions of sRNAs in *S. erythraea*, including sRNA-mediated regulation of erythromycin biosynthesis.

## Introduction

*Saccharopolyspora erythraea* is a gram-positive filamentous bacterium that was originally identified as *Streptomyces erythraeus*, but later assigned to the genus *Saccharopolyspora* [1]. *S. erythraea* is a prolific producer of a large number of secondary metabolites with biological activities, including erythromycin, a property that is probably correlated to the competitive environment in which the bacteria live. Studies focused on secondary metabolites of *S. erythraea* may provide a better understanding of the biosynthesis and regulation of erythromycin.

The commercial importance of erythromycin has fostered intensive research into its biosynthesis, and genetic engineering of the pathways involved in its biosynthesis is a promising approach for enhancing the production of potentially-valuable analogs of polyketide secondary metabolites [2]. The increasing interest in erythromycin production has revived efforts to increase the productivity of bacterial strains. Traditionally, wild-type actinomycete strains are subjected to multiple rounds of random mutagenesis and selection to obtain mutants that overproduce the desired secondary metabolite for industrial production. In 2007, the genome of *S. erythraea* was completely sequenced and annotated [3], thereby facilitating the expedited optimization of such actinomycete strains for production. In 2008, with the establishment of the 454/Roche GS FLX sequencing method, we completed, for the first time, the whole genome sequencing of the industrial erythromycin-producing *Saccharopolyspora* strain HL3168 E3 (hereafter referred to as the E3 strain) by using traditional mutagenesis methods. Annotation of the 8199523-bp genome was completed in 2009. Thereafter, 7195 open reading frames (ORFs) were predicted using the prediction software of Glimmer and GeneMark [4,5] and 50 tRNAs and four groups of rRNAs were predicted with the tRNA-scan software [6], adding up to a total of 12 rRNAs. In addition, 186 mutations in the protein-encoding genes were identified, thus building a solid foundation for the assignment of *S. erythraea* gene functions. The transcriptional regulation of metabolic processes and the control of secondary metabolism have been studied extensively to date; however, little is known about the extent and importance of post-transcriptional regulation in this organism. Within the last few years, small non-coding RNAs (sRNAs) have been implicated as important post-transcriptional regulators in a variety of adaptive cellular and developmental processes, as well as during virulence in bacteria [7-10]. Studies have also suggested that sRNAs play an important role in the regulation of secondary metabolites.

sRNAs have been known to be present in bacteria since the early 1970s, but a full appreciation of their prevalence and impact on various biological regulatory processes has only been revealed in recent systematic genome-wide searches for these molecules and their genes [11-13]. sRNAs are non-coding, functional, or regulatory RNAs, which are different from messenger (mRNA), transfer (tRNA) or ribosomal (rRNA) RNAs. Although they cannot be translated into proteins, they can regulate gene expression at more than one level, (such as at the RNA processing, modification, and stability steps, or at the transcriptional and translational levels) and can also influence protein stability and transfer effects [14]. sRNAs vary in their size (from ^~^50- to 600 nt long), structure, and function, and they are usually not translated into proteins (there are some exceptions). In bacterial metabolism, sRNAs are involved in a growing number of regulatory pathways in response to environmental changes [15].

In recent years, sRNAs have attracted great interest as ubiquitous regulators in all kingdoms of life. However, a smaller number of sRNAs have been identified and characterized in bacteria than in eukaryotes. Since the first report on a fully sequenced bacterial genome, an increasing number of genome-wide computational screens for sRNAs in microorganisms have been conducted [16]. In some cases, the mode of action and the mechanisms employed by bacterial sRNAs are fairly well understood [17,18]. These previous studies have suggested that a few sRNAs regulate target genes by binding to (regulatory) proteins, but most other sRNAs act as antisense RNAs on *cis*-encoded or *trans*-encoded mRNAs. Functional studies reveal that a large subset of these sRNAs act by an antisense mechanism, usually around the translation start sites of the corresponding targets, to modulate gene expression at the post-transcriptional level.

To date, many *in silico* strategies have been used to identify a wealth of sRNA candidates in various organisms, from bacteria [19] to humans [20], some of which were subsequently validated experimentally and considered as novel sRNAs [21]. On the basis of previous research, Herbig et al. [22] developed the nocoRNAc software, which, together with the RNAz software used in sRNA prediction in *Streptomyces coelicolor*, has been used to detect more than 800 sRNAs.

Deep sequencing has recently emerged as a powerful tool for the identification of sRNAs [23-27]. Vockenhuber et al. [28] presented the analysis of the primary transcriptome of *S. coelicolor* M145 by using a differential RNA-sequencing (dRNA-seq) approach and the 454 sequencing technology. For *S. erythraea*, Marcellin et al. [29] used a combination of small RNA sequencing and long RNA-seq data and identified 190 putative ncRNA in intergenic genomic regions.

sRNA gene identification and functional studies have been conducted in model organisms, including *E. coli* and *Salmonella Typhimurium* [8,18,19,30]. In addition, prediction and partial validation of the existence of sRNAs in *Streptomyces* have been described in several reports [16,28,29,31-33]. In particular, Davide [16] showed that reversal of the overexpression of a *cis*-encoded antisense sRNA in the glutamine synthetase I gene of *S. coelicolor* resulted in a decrease in growth, protein synthesis, and antibiotic production. *S. erythraea* is closely related to *S. coelicolor*. Therefore, the study of sRNA in *S. erythraea* will help elucidate the regulatory mechanisms operative in secondary metabolite biosynthesis, which in turn, will enable the use of genetic engineering methods to regulate and increase the production of erythromycin, the major secondary metabolite.

In this study, we focused on the identification of sRNAs associated with secondary metabolite regulation in *S. erythraea*. Using a variety of bioinformatics tools and deep-sequencing technology, sRNAs in *S. erythraea* were systematically screened and analyzed. We believe that the findings of this study will add to our current knowledge of the regulatory network of secondary metabolism, which can be applied to improve the production and activity of erythromycin.

## Materials and Methods

### Multi-sequence alignment using MAUVE

Genome-wide prediction of sRNA by RNAz is based on the structure conservation index (SCI). Consequently, a genome comparison method named progressive MAUVE [34], which identifies conserved genomic regions, rearrangements and inversions in conserved regions, and the exact sequence breakpoints of such rearrangements across multiple genomes, was used to perform the multi-sequence alignment. In order to obtain the conserved genomic regions for RNAz analysis, the genomes of actinomycetes, including *Mycobacterium tuberculosis* [RefSeq: NC_000962], *Nocardia farcinica* [RefSeq: NC_006361], and the model actinomycete *Streptomyces coelicolor A3* (2) [RefSeq: NC_003888.3], and *Saccharopolyspora erythraea* [RefSeq: NC_009142], were aligned with MAUVE, and then the resultant MAF file was used as the input for RNAz.

### Prediction of sRNAs *in silico*

The RNAz program was used for the genome-wide prediction of sRNA loci [35]. In this program, a sequence alignment was used as input and classified as “structural RNA” or “OTHER”. The prediction approach of RNAz is based mainly on the following premises: First, the structure of functional sRNA sequences is much more stable than that of non-functional sRNA sequences. Second, a lower SCI is considered as an indicator for non-coding RNAs. The structure of functional RNAs is usually more conserved among related species than that of other sequences.

The multiple sequence alignment processed by MAUVE was input into the RNAz software package. To detect sRNAs of different sizes, several runs of RNAz were performed with different settings for the window size, i.e., 60, 80, 100, 120, and 160 nt. The step size was set to 20 nt. After application of RNAz, overlapping windows that had been classified as “structural RNA” were joined to predict sRNA loci. The predicted sRNA loci were then used as input for nocoRNAc [22]. In brief, the nocoRNAc package calculated the Stress Induced Duplex Destabilization (SIDD) profile to predict the SIDD sites along with the results of the terminator predicted by TransTermHP, which were assigned to the sRNA regions. Furthermore, the nocoRNAc package predicted sRNA transcripts based on the results of RNAz, in combination the SIDD profile and terminator information. Analysis of the sRNA target and RNA-RNA interaction prediction were processed with the Vienna RNA package [36].

### Cultivation of *S. erythraea*

*S. erythraea* NRRL2338 (ATCC11635) (hereafter referred to as the M strain) was used as the reference wild-type strain, and the E3 strain, which is used in the industrial production of erythromycin, was used as the contrast or test strain. Both strains were cultivated under the following conditions: 10^8^ spores/50 mL of medium were pre-germinated and cultured in YEME medium (3 g yeast extract, 5 g tryptone, 3 g malt extract, 10 g glucose, and 2.5 M MgCl_2_-6H_2_O per liter of distilled H_2_O) with glass beads (2 g/50 mL) at 30°C under continuous shaking (200 rpm) to the end of the exponential phase (72 h). One milliliter of the culture was harvested by centrifugation at 8,500 rpm at 4°C for RNA extraction. 

### RNA isolation

Total RNA was isolated after culturing for times (24, 48, and 72 h) by using the RNeasy Mini Kit (QIAGEN) according to the manufacturer’s instructions. The RNA sample harvested at the 72-h time-point was used for deep-sequencing analysis. One hundred micrograms of total RNA was incubated with 30 U of Turbo DNase (Ambion) for 1 h to remove residual DNA, and then subsequently precipitated and suspended in 50 μL of water. The average concentration obtained was 1-1.5 μg/μL, and the quality of the recovered RNA was assessed by electrophoresis on 1% agarose gel followed by GoldView staining and UV transillumination.

### Preparation of cDNA library and sequencing

Total RNA that was harvested from *S. erythraea* (strains E3 and M) grown in a liquid-rich medium (YEME) for 72 h until the end of the exponential growth phase, at which time secondary metabolism is usually initiated, was analyzed by deep sequencing according to Ovation Prokaryotic RNA-Seq System kit protocol (NuGEN Technologies, Inc., San Carlos, CA, USA). Two independent replicates of RNA samples collected from each strain were analyzed. Briefly, first- and second-strand cDNA synthesis were performed with selective priming to enrich non-rRNA transcripts from bacterial total RNA inputs. cDNA was purified using the QIAquick PCR Purification Kit (Cat. # 28104, 28204; QIAGEN), according to the manufacturer’s protocol. Following end repair, the double-stranded cDNA was found to be compatible with NuGEN’s Encore™ NGS Library Systems that allow easy sample multiplexing within a single-end, transcriptome sequencing format.

Double-stranded cDNA was processed for RNA-Seq by using the Illumina Genomic DNA Sample Prep Kit (Illumina) according to the manufacturer’s instructions. Sequencing was carried out by running 2 × 100 cycles on Hi-Seq 2000, and the deep sequencing data are available in the NCBI GEO database (accession number: GSE48887).

For evaluating the differential expression of the same sRNA candidate in the M and E3 strains, the results of RNA-Seq of every sRNA candidate was normalized and compared in M and E3 with the Reads per Kilobase per Million mapped reads (RPKM) index [37], where RPKM is calculated as follows:

$$\text{RPKM}=\frac{\text{transcription\_reads}}{\text{transcription\_length}\times \text{total\_assembly\_reads\_in\_run}}\times {\text{10}}^{\text{9}}$$

Where “transcription_reads” are the reads that cover the whole unique transcript; “transcription_length” is the whole unique transcript length; and the “total_assembly_reads_in_run” are the number of reads covering all reads involved in the splitting joint.

### qRT-PCR

Total RNA was harvested at different stages of cell growth. cDNA synthesis was carried out using the PrimeScript RT reagent Kit (TAKARA Biotechnology [Dalian]). First, residual genomic DNA was eliminated from the sample with a gDNA eraser at 42°C for 2 min, followed by reverse transcription at 37°C for 15 min and 85°C for 5 s. cDNA was used to analyze sRNA expression by real-time quantitative PCR (qPCR) in a Bio-Rad cfs 96 by using SsoAdvanced SYBR Green Supermix (Bio-Rad, USA). PCR reaction mixtures were denatured at 95°C for 30 s, followed by 42 cycles at 95°C for 5 s and 57°C for 30 s, with data collection at 57°C. Amplification of the appropriate product was confirmed by melting curve analysis following amplification. Raw threshold cycle (Ct) values were calculated using the Bio-Rad CFX Manager software v1.6 using automatic baseline settings. Thresholds were set in the linear phase of the amplification plots. The primers are listed in Table 1. Expression of the sRNA genes at different growth phases (24, 48, and 72 h) were calculated relative to the calibration sample and an endogenous control (16S rRNA) to normalize the sample input amount by using the formula 2^-ΔΔCt^ (ΔCt = C_t_ gene of interest-Ct endogenous control) [38]. The experiment was repeated at least 3 times. 

**Table 1:** Primers used for RT-PCR to amplify six sRNA.

Primer ID	Sequence	Primer length (nt)	Products
sernc292F	TCTTTTTGTCCTCGGTTGCC	20	103
sernc292R	ATCCTCACGCGACGCAATG	19	
sernc293F	TGGTGCAACACAGGTACGG	19	115
sernc293R	ACACCGACCGGCTTGATG	18	
sernc350F	ACGGGAAGGTCAACAAGATCG	21	130
sernc350R	TTGATGGTGTACTCCCAGTCG	21	
sernc351F	TGGACAACCTGATCCAGAACC	21	148
sernc351R	ATTTCCGGCGTGTCGAACAC	20	
sernc361F	TGGCTTCGGCTTTCTGAATC	20	134
sernc361R	CGGATGTCGTTACCAAAGCAC	21	
sernc389F	ACCTTGTTCTTGGCTTTGCG	20	118
sernc389R	AGAAGATCGCGAACCCGAAG	20	
16srRNAF	CATTGCTGCGGTGAATAC	18	151
16srRNAR	GGCTACCTTGTTACGACTT	19	

## Results

### *In silico* prediction of sRNA of *S. erythraea*

**Figure 1:**
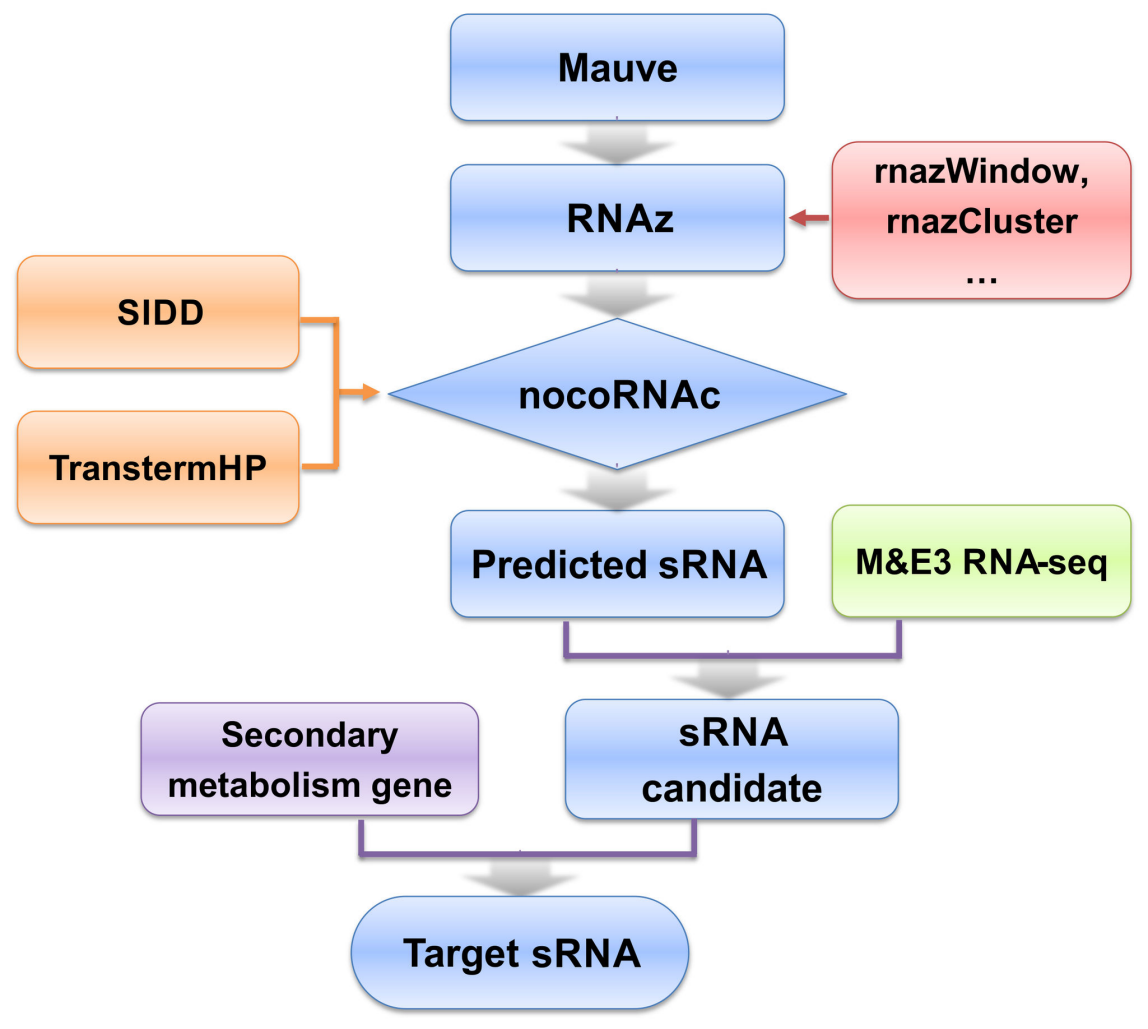
Flowchart of the procedures for identifying sRNA genes.

sRNA candidates were predicted using the following strategy (Figure 1). Alignment of the genomes of *S. erythraea*, *M. tuberculosis*, *N. farcinica*, and *S. coelicolor* was generated using MAUVE, which included all conserved genomic regions in the four genomes. The results were then used as input for the RNAz package, which calculated the minimum free energy (MFE) and SCI. RNAz predicted 788 sRNA loci for the reference organism *S. erythraea*. Finally, combining the SIDD and TransTermHP data, nocoRNAc was used for the transcript models to generate the final list of candidates. Of the 788 loci analyzed, nocoRNAc predicted 647 sRNA transcripts, of which 382 are complementary to protein-coding regions (Table S1). Two hundred and sixty-five predicted transcripts are located in intergenic regions (Table S2). The length of the predicted sRNAs ranged from 30 to 837 nt, with an average length of 293 nt. Six candidate sRNAs were found to be associated with 3 gene clusters (*tpc3*, *pks6*, and *nrps5*) that are involved in secondary metabolite biosynthesis (Table 2). The four *cis*-encoded antisense sRNAs sernc293, sernc350, sernc351, and sernc389 were complementary to their targets SACE_3034, SACE_3976, SACE_3977, and SACE_4573, respectively. The targets (*trans*-antisense) of two sRNAs (sernc292 and sernc361) were predicted with RNApredator [39] to be SACE_7209, which encodes heat shock protein (HSP-70 cofactor) and SACE_4129, which encodes a tetracycline-repressor (TetR) family transcriptional regulator, respectively.

**Table 2:** Gene clusters for secondary metabolites production and the number of regions predicted sRNA.

Clusters	Gene number	Probeset SACE ID	Regions predicted sRNA
**Terpenes**			
*tpc1 (geo1)*	1	3187	
*tpc2*	3	3721–3723	
*tpc3 (geo2)*	4	3976–3979	3976; 3977
*hop*	5	4327–4331	
*tpc4*	10	4645–4654	
*tpc5 (geo3*)	4	4906–4909	
**Polyketides**			
*pfa*	11	0018–0028	
*ery*	21	0712–0721, 0723–0734	
*rpp*	4	1241–1244	
*pks1*	6	2342–2347	
*pks2*	4	2628–2631	
*pks3*	16	2864–2879	
*pke*	18	4128–4145	4129-4130
*pks4*	6	4302–4307	
*pks5*	8	4471–4478	
*pks6*	12	4567–4578	4573
*pks7*	4	5306–5309	
*pks8*	1	5532	
**Non-ribosomal peptides**			
*nrps1*	7	1304–1310	
*nrps2-pks*	4	2618–1622	
*nrps3*	12	2692–2703	
*nrps4*	5	3013–3017	
*nrps5*	11	3029–3039	3032-3033; 3034
*nrps6*	7	3223–3229	
*nrps7*	18	4275–4292	
*Total*	202	Targeted	

### Deep sequencing and characterization of sRNAs

RNA-Seq analyses were performed on two independent replicate RNA samples collected from *S. erythraea* strains M and E3 that were grown to stationary phase. cDNA was generated from mRNA-enriched total RNA preparations from each strain and sequenced using the Illumina Hi-Seq 2000. All data are available at GEO (GSE48887). In accordance with the *in silico* prediction, the deep-sequencing information of sRNA candidates was explored (Tables S1 and S2). Several sequences were duplicated, since every transcript was sequenced several times. The duplicates were filtered using bioinformatics software; the largest number of unique reads of sRNA candidate in E3 was for sernc350 (1063 reads), while in M, it was for sernc113 (743 reads). To evaluate the difference in expression of the same sRNA candidate in E3 and M, the RPKM index was calculated. The results indicate that the greatest ratio for a sRNA candidate was obtained for sernc350, whose expression in strain E3 is up to 30-times higher than that in strain M.

### Extraction of candidate sRNAs related to secondary metabolism of *S. erythraea*

**Figure 2:**
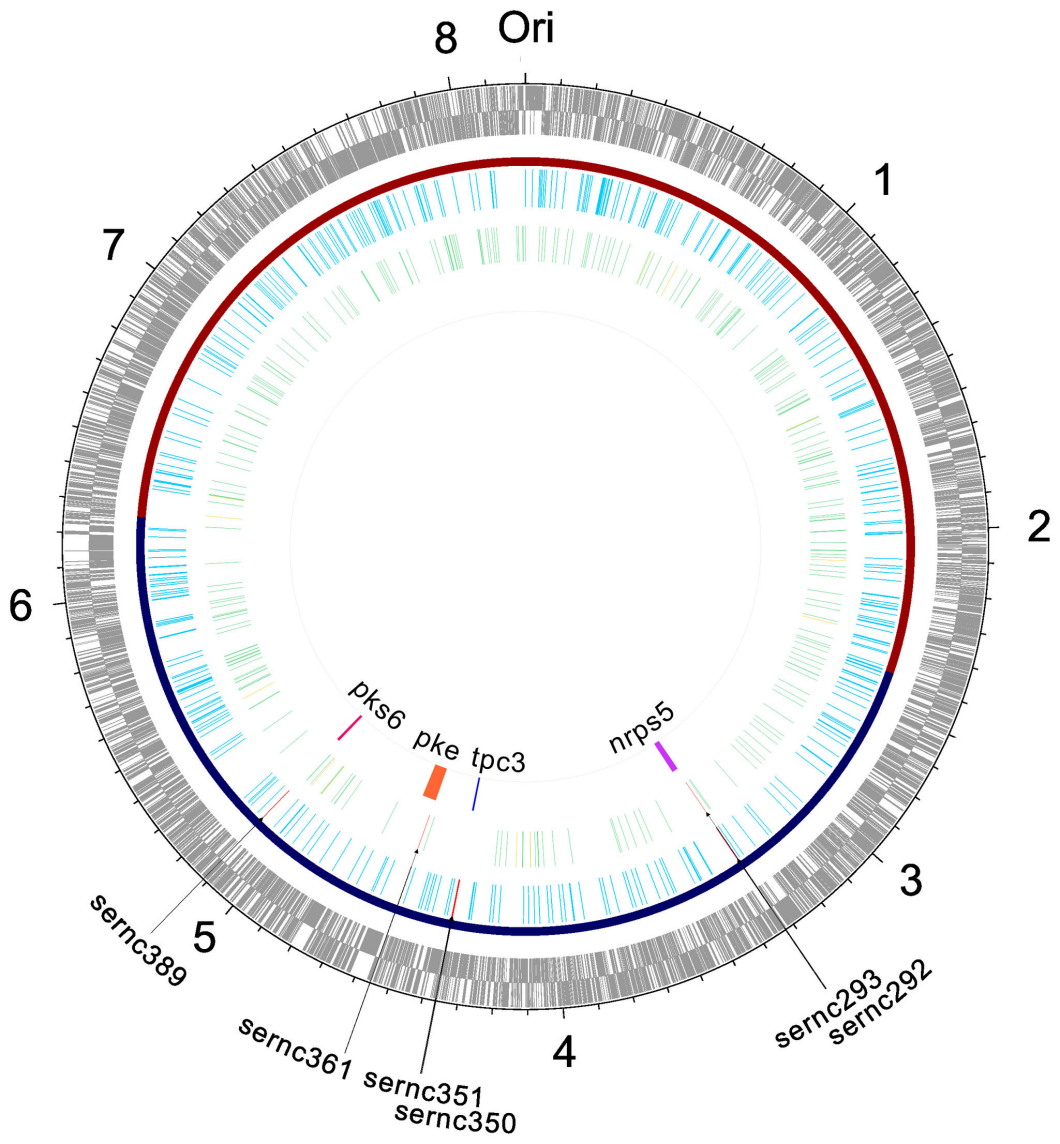
Schematic representation of the genomic positions of sRNA genes. Circles 1 and 2 (from the outside in), all genes represented through reverse and forward strand, respectively; circle 3, the core (red) and noncore (blue) genome regions; circle 4, cis-encoded antisense sRNA predicted in this study, in which, the sRNA related to secondary metabolism colored by red; circle 5, trans-encoded antisense sRNA predicted in this work, in which, the sRNA overlapped with previous study (Marcellin et al., 2013) colored by yellow, and the sRNA related to secondary metabolism colored by red; circle 6, gene clusters correlated with the sRNA correlated with secondary metabolism.

**Figure 3:**
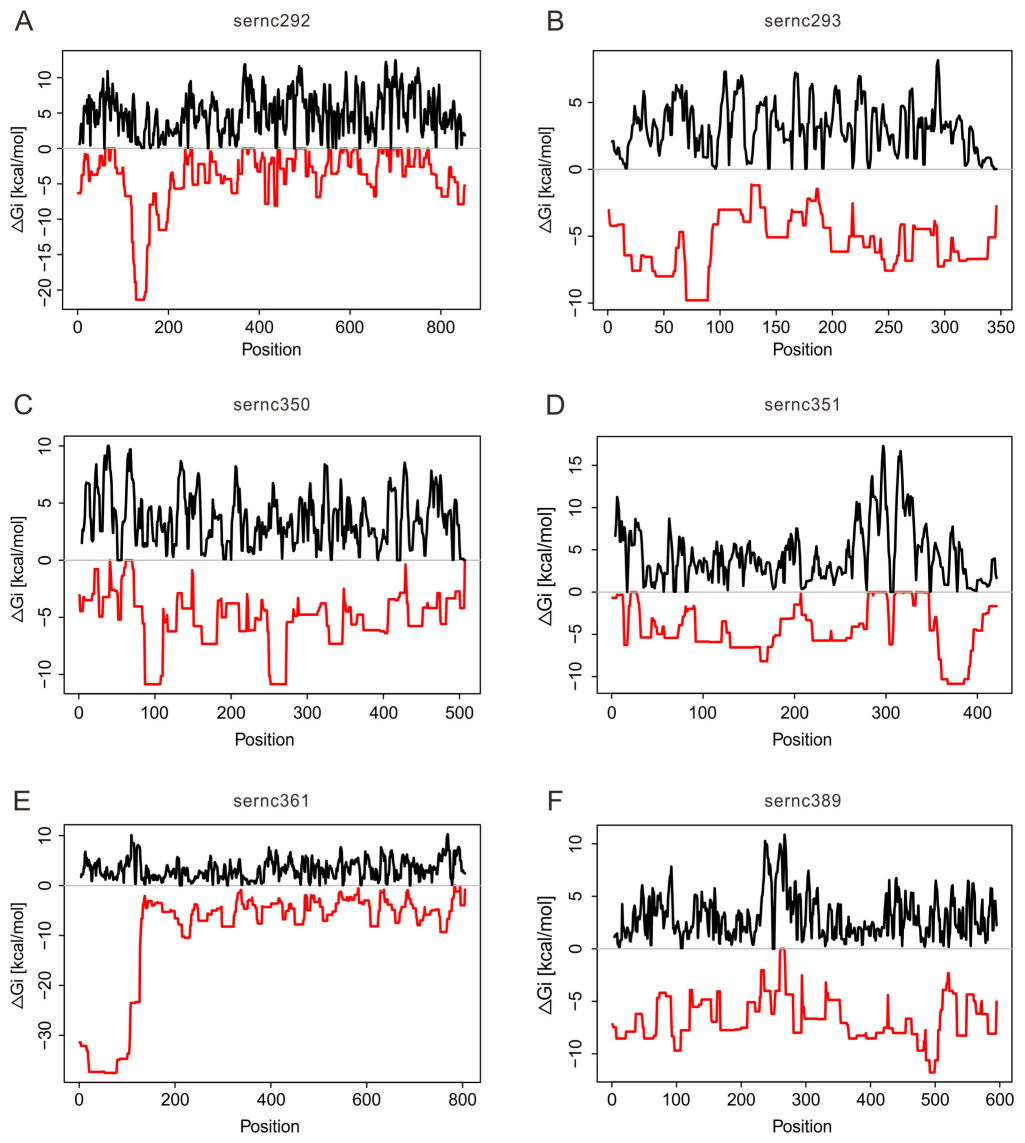
RNA-RNA interactions of the six sRNAs and their targets. The sRNA and its corresponding target are shown for the following sRNA-target pairs: (A) sernc292 and SACE_7029, (B) sernc293 and SACE_3034, (C) sernc350 and SACE_3976, (D) sernc351 and SACE_3977, (E) sernc361 and SACE_4129, and (F) sernc389 and SACE_4573. Black line indicates the local opening energies for both molecules and the red line shows interaction energies. The best interaction site of two molecules could be analyzed by computing the interaction energies.

In *S. erythraea*, 202 genes in 25 gene clusters are directly associated with secondary metabolites (Table 2). Six candidate sRNAs associated with secondary metabolism genes were identified in the present study, of which four candidate sRNAs (sernc293, sernc350, sernc351, and sernc389) were predicted to interact with secondary metabolism genes by base-pairing. Two sRNAs (sernc292 and sernc361) were located in the intergenic regions between SACE_4129 and SACE_4130 and SACE_3032 and SACE_3033, respectively (Figure 2). The corresponding targets are SACE_7029 and SACE_4129, respectively. MFE-based predictions of the secondary structures of the six sRNAs were performed using the Vienna RNA package [40] (Figure S1). Accessibility of the sRNA with its corresponding target region was also analyzed (Figure 3).

### Experimental validation of candidate sRNAs

**Figure 4:**
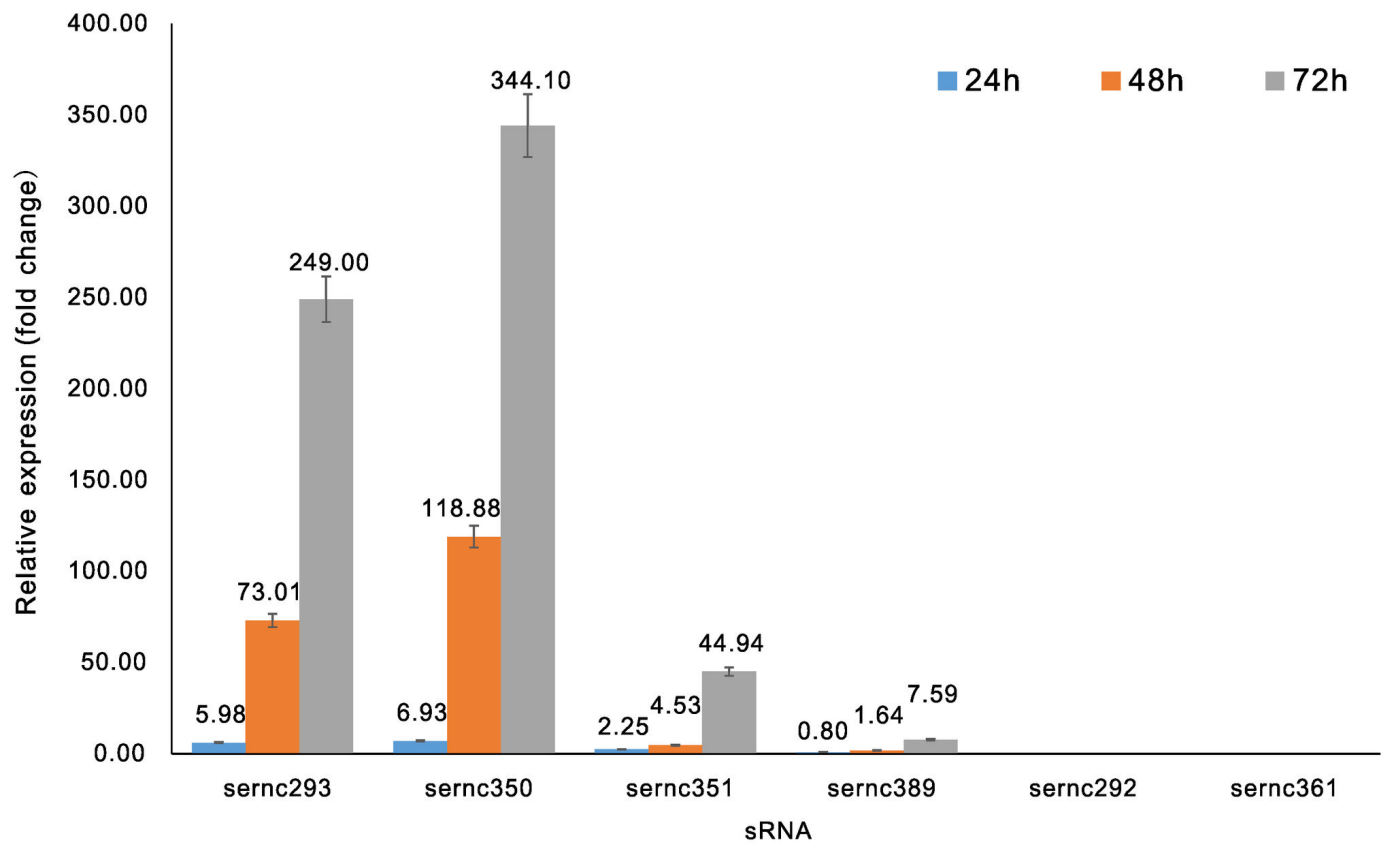
Relative expression analysis of sRNAs by real-time RT-PCR. The differential expression of the six sRNAs was analyzed using qRT-PCR in strains M and E3 at 24 (blue), 48 (yellow), and 72 h (gray).

QRT-PCR was performed to assess the expression of the six novel sRNA (from candidate sRNAs sernc292, sernc293, sernc350, sernc351, sernc361, and sernc389) related to secondary metabolism, which were predicted and identified in this study, and the results were standardized to the 16s rRNA gene (SACE_8101). Total RNA was isolated from *S. erythraea* after 24, 48, and 72 h of culture, representing the three growth phases. Samples with a Ct > 40 were considered negative. The results indicated that differential expression of sernc293, sernc350, sernc351, and sernc389 at the three time points were detected in both M and E3 strains, in which, the four sRNAs showed higher differential expression at 72 h with 249-, 344-, 44.94-, and 7.59-fold change than that observed at 24 h (5.98-, 6.93-, 2.25-, and 0.80-fold change, respectively) and at 48 h (73.01-, 118.88-, 4.53-, and 1.64-fold change, respectively; Figure 4). In contrast, no expression was detected in the other two sRNAs sernc292 and sernc361. 

## Discussion

*S. erythraea* is a gram-positive, spore-forming bacterium. The molecular mechanisms that are altered with the traditional mutation and screening approaches during the improvement of antibiotic-producing microorganisms are still poorly understood in this bacterium, although this information is essential to the design of rational strategies for industrial strain improvement. Delineation of the regulatory network of secondary metabolites could facilitate the understanding of the regulatory mechanism of secondary metabolism, including the biosynthesis of erythromycin. In this study, a systematic method for the prediction and analysis of sRNAs of *S. erythraea* has been reported, with the objective of providing a better understanding of the sRNA-mediated regulatory network of secondary metabolites.

This study utilizes *in silico* and deep-sequencing technologies to predict sRNAs of *S. erythraea*. Through *in silico* prediction methods, the RNAz software package was used to perform a genome-wide search for sRNA and the MFE and SCI of every transcript were evaluated. Most bacterial sRNAs are transcribed from their own promoters, and transcription most often terminates at a strong Rho-independent terminator. This property was also considered for the prediction/identification of sRNAs. On the basis of the RNAz results, in combination with the promoter and terminator information, the nocoRNAc package was used to filter and analyze the sRNAs predicted by RNAz. These profiles have been applied successfully in the model actinomycete *Streptomyces coelicolor* A3(2) [22]. In the present study, we used deep-sequencing to analyze the difference of sRNAs predicted *in silico* in strains *S. erythraea* M and E3, which could facilitate the prediction of sRNA related to secondary metabolism.

Most previous studies mainly predicted and analyzed sRNAs only in intergenic regions (IGRs) [41-43]. However, several recent studies have demonstrated that the sRNAs partially encoded on the non-coding strands of ORFs play an important role in regulating metabolic pathways at the post-transcriptional level [44,45]. Similar results were observed in this study. Through RNAz and nocoRNAc modeling, 647 candidate sRNAs were predicted, in which 382 (approximately 59%) sRNAs were antisense to mRNA through base-pairing and 265 (approximately 41%) candidate sRNAs were found in intergenic regions. In the *trans*-encoded antisense sRNA, 14 sRNAs overlapped with previous study [29] of other *S. erythraea* mutant (Table S2, Figure 2), but all of them were not related to secondary metabolism. 

A remarkable feature of the E3 strain is its ability to biosynthesize secondary metabolites more efficiently than that of strain M. This enhanced biosynthesis requires the regulation of bacterial gene expression to adapt to environmental fluctuations. To identify sRNAs that are directly related to secondary metabolism, we focused on the sRNAs that were associated with the secondary metabolism gene clusters. From the results of the *in silico* prediction and deep-sequencing analysis, six sRNAs associated with secondary metabolism genes were identified, of which sernc293, sernc350, sernc351, and sernc389 were encoded on the complementary DNA strands of annotated ORFs (*cis*-antisense). Antisense RNAs are diffusible regulatory RNAs that bind to the complementary sequences of mRNAs and regulate their biological functions at the post-transcriptional level [46]. 

To validate the function of the predicted sRNAs, differences in the expression of the six sRNAs identified in strains M and E3 at three different time points (24, 48, and 72 h) were analyzed using qRT-PCR. Previous studies have shown that approximately 40% of the genes exhibited an expression profile that strongly correlated with the time course in *S. erythraea* [47]. Our experimental results show that the expression of four antisense transcripts (sernc293, sernc350, sernc351, and sernc389) was detected using qRT-PCR at all the three time points in strain E3 and M, but the expression was higher in E3 than that in M.. No significant expression of sernc292 and sernc361 was detected at these three time points in either strain. However, we were not able to determine whether these are false-positive predictions or whether they are or can be expressed under different conditions.

The genomic location and synteny of sRNAs might help elucidate the target genes and functions of these sRNAs. The results show that sernc293, sernc350, sernc351, and sernc389 are complementary to SACE_3034, SACE_3976, SACE_3977, and SACE_4573, respectively. The coding product of SACE_3034 is an ABC Fe^3+^ transporter-binding protein. Ferrous ions are essential micronutrients for almost all living organisms. Iron is present in the active site of several enzymes involved in major biological processes such as respiration, DNA biosynthesis, tricarboxylic acid (TCA) cycle, gene regulation, and production of metabolites. However, iron also becomes toxic at high concentrations by reacting with hydrogen peroxide to generate highly reactive oxygen species (ROS). These ROS damage nucleic acids, proteins, and cellular membranes. Mainly for this reason, many organisms have developed strong homeostatic systems that maintain intracellular Fe^2+^ concentrations [48]. Recent studies have demonstrated that sRNAs have key roles in the bacterial response to stress. For example, a small RNA in *E. coli*, RyhB, was found to down-regulate a set of iron-storage and iron-utilizing proteins under limited iron availability; RyhB was negatively regulated by the ferric uptake repressor (Fur) protein [49]. sernc293 is located at the 5’-end and around the translation start site of SACE_3034, and it likely participates in the regulation of iron transport by pairing with target mRNA. The results of deep-sequencing demonstrated that the expression of sernc293 in strain E3 was higher (approximately six-fold) than that in strain M; similarly, qRT-PCR analysis results demonstrated that the expression of sernc293 in strain E3 was higher (up to 249-fold) than that in strain M, at the 72-h time point. These data suggest that sernc293 may plays an important role in strain E3 in the regulation of secondary metabolism.

SACE_3976 encodes cyclic nucleotide-binding-domain protein that usually binds to secondary messengers. Since the discovery of cAMP in the 1950s, the role of these secondary messengers in mediating cellular function has become a major theme of research in biology. Sernc350 regulates SACE_3976 by base-pairing to the 5’-end and around the translational start site. SACE_3976 encodes a catabolite activator protein (CAP) family transcription factor that is associated with the *tpc3* gene cluster, and regulate terpene metabolites. Deep-sequencing results shows that the expression of sernc350 in strain E3 was higher (up to 30-fold) than that in strain M. In addition, qRT-PCR analysis showed that high differential expression of sernc350 was detected between strain E3 and M at all the three time points. These data indicate that sernc350 is vital for terpene metabolic regulation in strain E3. 

SACE_3977, similar to SACE_3976, also belongs to the *tpc3* cluster, although it encodes terpene synthase. In the last 5-10 years, it has become evident that terpenes are produced by numerous bacteria, especially by soil-dwelling gram-positive organisms such as *Streptomyces* spp. and other *actinomycetes*. Some microbial terpenes, such as geosmin, have been known for over 100 years [50]. Previous studies have suggested that geosmin synthesis is directed by the *tpc3* cluster [47]. Consequently, sernc350 and sernc351 may be correlated to the regulation of geosmin synthesis. 

The target of sernc389 is SACE_4573, which encodes an IS200 transposases. IS200 transposases originally identified in *Salmonella Typhimurium* LT2, are present in many bacteria and archaea, and are distinct from other groups of transposases [51]. Meanwhile, SACE_4573 belongs to *pks6* cluster, which encodes genes for polyketide biosynthesis. In a previous study, Peano et al. [52] analyzed the difference of *S. erythraea* NRRL2338 and an erythromycin over-producing strain (Px) at the genomic and transcriptional levels. The results indicate the pks6 cluster was up-regulated in Px strain, which is consistent with the results obtained in this study. Accordingly, sernc389 may improve erythromycin biosynthesis through its role as the metabolic regulator of polyketide biosynthesis, but the actual mechanism is still unclear.

A previous study demonstrated that *S. erythraea* undergoes a metabolic switch in its lifecycle, followed by a secondary growth phase [29]. In the present work, *S. erythraea* exhibited a distinct high differential expression of the four *cis*-encoded antisense RNA at 72 h, which is probably because of the time point behind the metabolic switch.

The principal findings of this study is that sRNAs in the regions close to the genes related to secondary metabolism in *S. erythraea* were successfully predicted and identified in this study. Nonetheless, our understanding of the targets and regulatory functions of sRNAs is still limited. In addition, experimental validation of the candidate sRNAs is needed. Therefore, our future work will focus on revealing the regulatory network of sRNAs in *S. erythraea*. We believe that a better understanding of the sRNA-target interaction will help elucidate the complete range of functions of sRNAs in *S. erythraea*, including the mechanism(s) through which sRNAs regulate the biosynthesis of erythromycin.

## Supporting Information

Figure S1 **The MFE-based predicted secondary structures of the six sRNAs.** The MFE stem-loop secondary structure of the six sRNAs of *S. erythraea*, as predicted by the Vienna package.
(TIF)

Table S1 **Overview of *cis*-encoded antisense RNAs.**
(XLS)

Table S2 **Overview of *trans*-encoded antisense RNAs.** The sRNA overlapped with previous study (Marcellin et al., 2013) to be highlight by yellow color.
(XLS)
